# The Estimation of Genetic Parameters for Longevity According to Lactation Period Using a Multiple Trait Animal Model in Korean Holstein Cows

**DOI:** 10.3390/ani12060701

**Published:** 2022-03-11

**Authors:** Jiseob Shin, Jaegu Lee, Juhyun Cho, Changgwon Dang, Taejeong Choi, Changhee Do, Jungjae Lee, Seokhyun Lee

**Affiliations:** 1 Dairy Cattle Improvement Center of NH-Agree Business Group, NACF, Goyang 10292, Gyeonggi-do, Korea; nicejiseob@naver.com (J.S.); dciccho@hanmail.net (J.C.); 2 National Institute of Animal Science, RDA, Cheonan 31000, Chungcheongnam-do, Korea; jindog2929@korea.kr (J.L.); gkgkgki@korea.kr (C.D.); choi6695@korea.kr (T.C.); 3 Division of Animal and Dairy Science, College of Agriculture and Life Sciences, Chungnam National University, Daejeon 34134, Chungcheongnam-do, Korea; ivando@cnu.ac.kr; 4 Department of Animal Science and Technology, College of Biotechnology and Natural Resources, Chung-Ang University, Anseong 17546, Gyeonggi-do, Korea

**Keywords:** longevity, Korean, Holstein

## Abstract

**Simple Summary:**

South Korea dairy farmers are less interested in improving milk production-related traits for various reasons, including the milk production quota system. Consequently, they have become more interested in improving traits associated with longevity. Therefore, the purpose of this study was to analyze the culling patterns and survival rates of Korean Holstein cows and evaluate genetic characteristics related to parity and longevity of each lactation by using test data collected in South Korea. This study presents evidence that individuals’ genetic backgrounds differ with respect to differences in the individuals’ survival periods. Three lactation periods in each parity were split according to lactation days: 0–90 days (1), 91–299 days (2), and 300 days to the next calving (3). The heritability of the first, second, and third parities were 0.020, 0.028, and 0.039, respectively. In all parities, the heritability in late-lactation was higher than that in early- and mid-lactations. Most genetic correlations for survival in the first parity were higher than those in the second and third parities. The genetic correlation in the same lactation in the first and second parities were lower than those in the intervals in the second and third parities. The results of this study may serve as a basis for developing a more accurate model for evaluating longevity traits in South Korea.

**Abstract:**

Longevity is closely related to the survival rate of dairy cattle and refers to the period during which the cow has economic value, from first calving to culling. The purpose of this study was to analyze the culling patterns and survival rates of Korean Holstein cows and evaluate genetic characteristics related to parity and longevity of each lactation by using the test day milk yield collected in South Korea. The performance data of the dairy cattle were collected from 2004 to 2019 by the Nonghyup Dairy Cattle Improvement Center. The collected 1,702,304 records were used as pedigree data through the Korea Animal Improvement Association. The lactation period was divided into early-lactation (0–90 days: L1.1, L2.1, and L3.1), mid-lactation (91–299 days: L1.2, L2.2, and L3.2), and late-lactation (300 days-next parity: L1.3, L2.3, and L3.3). The heritability of longevity for the first, second, and third parity was 0.020, 0.028, and 0.039, respectively. In all parities, the heritability in late-lactation was higher than that in early- and mid-lactation. Most genetic correlations for survival in the first parity were higher than those in the second and third parities. The results of this study may serve as a basis for developing a more accurate model for evaluating longevity traits in South Korea.

## 1. Introduction

The purpose of the Dairy Cattle Improvement Project of South Korea is to increase the income of farmers by improving their productivity and enhance the competitiveness of sire in South Korea. Domestic dairy farmers are less interested in improving milk production-related traits for various reasons, including the milk production quota system. Consequently, they have become more interested in improving traits associated with longevity, which have been introduced in regions that have made the most advancements in dairy technology such as North America.

Longevity is closely related to the survival rate of dairy cattle and refers to the period during which the cow has economic value, from first calving to culling. In general, longevity is a desirable trait affecting dairy farmers’ profitability [1,2]. The mean yield of the herd could be improved by an increase in longevity [3,4].

In the dairy industry, it has also been reported that genetic evaluation for longevity has been conducted in all advanced countries [5]. Longevity often defines dairy cattle’s lifetime using one genetic trait [6,7,8]. However, several studies have shown that longevity is genetically different from lactation to lactation [9,10,11]. Other studies reported that various diseases and reproductive problems caused the culling of dairy cattle, and this varied by the longevity of each lactation period [12,13,14,15]. These results imply that the genetic background for survival may differ in different periods during lactation in days [16]. This hypothesis can be supported by the different reasons for culling investigated by dairy farmers, and the type of culling depends on parity and lactation in days [17]. Roxström and Strandberg [18] found that cattle were culled for genetically different reasons. Moreover, Ducrocq [19] discovered a strong indicator suggesting that survival at later lactation in days was genetically distinct from that at early lactation, regardless of the number of lactations. Van Pelt, et al. [20], reported that the genetic background of survival was transforming with time using the definition of the survival trait based on the entire productive life, and evaluated the longevity genetic capacity using the survival status in each lactation.

Calculating the survival probability for each parity and lactation is possible in South Korea using the milking and culling records collected as test records. The survival lifetime can be explained by the different reasons for culling collected from dairy farmers this way, and the reasons for culling vary by parity and lactation in days [21]. Therefore, the purpose of this study was to evaluate genetic characteristics related to parity and longevity of each lactation by using the test data collected in South Korea.

## 2. Materials and Methods

### 2.1. Phenotypic Data

The performance data related to milk production and udder health of dairy cattle were collected from 2004 to 2019 by the Nonghyup Dairy Cattle Improvement Center. The 1,702,304 records collected through the Korea Animal Improvement Association were used as pedigree data.

The records of 190,773 cows were used for estimating genetic parameters after removing censored data, cases with unknown parent records, and data with less than five contemporary groups (herd-year). The genetic evaluation was using 298,975 records, including censored (190,773 records) and uncensored (108,202 records) data among the test records collected for breeding value estimation. The uncensored data refers to cases with known culling time. The censored data refer to the data that cannot measure the exact productive life because it is unknown if an individual has survived, or the exact date of culling is unknown. The detailed reasons for culling and records are in Supplementary Table S1.

The lactation period was divided into early-lactation (0–90 days: L1.1, L2.1, and L3.1), mid-lactation (91–299 days: L1.2, L2.2, and L3.2), and late-lactation (300 days-next parity: L1.3, L2.3, and L3.3).

When an individual survived in the subsequent lactation period from the current lactation period, the individual was coded as 1. If an individual did not move to the next lactation period because of culling or death, it was coded as 0. Moreover, for late-lactation cases (more than 300 milking days), if a test record existed for the subsequent parity, it was set to 1. If not, it was set to 0 (Table S2).

### 2.2. Analysis Model

The following animal model was used for estimating genetic parameters.

$$Y_{ijkl}=HY_{i}+Season_{j}+FCa_{k}+a_{ijkl}+e_{ijkl}$$

where, *Y* is the survival of each lactation (1 or 0), *HY* (herd-year) is the effect of the contemporary group, Season with four levels (Winter: December–February, Spring: March–May, Summer: June–August and Autumn: September–November) is the calving season effect, and *FCa* is the first calving age (<25 months of age: 0 and ≥26 months of age: 1), *a* is a random genetic effect, and *e* is a residual effect.

Although the contemporary group used for the genetic evaluation model of production traits and conformation traits is usually set to herd-year-season (HYS) and analyzed, this study used HY and season separately because the Korean dairy cattle population was too small to use HYS. A previous study also showed that the contemporary group of using HYS as a fixed effect and that of using HY and season as fixed effects had the best fit, respectively [22].

The multiple trait model that evaluates nine traits simultaneously demands a computer with high computation power. Therefore, because of computing power limitations, the parameter estimation was conducted by converting the nine traits (L1.1, L2.1, L3.1, L2.1, L2.2, L2.3. L3.1, L3.2, and L3.3) into 83 combinations of three traits. Afterward, the results were combined. The genetic parameters used in this study were estimated using the WOMBAT Program [23].

The genetic and phenotypic parameters of economic life by parity (G* refers to genetic parameters, and P* refers to phenotypic parameters) were calculated using the following equations.

$$G^{*}={W\prime \mathrm{GW}\;\mathrm{and}\;P}^{*}=W\prime \mathrm{PW}$$

where, W is an incidence matrix corresponding to each parameter and parity, W′ is a transpose of W matrix, whereas G* and P are genetic parameter matrices estimated in this study.

### 2.3. Breeding Value Estimation

When making a genetic variance–covariance matrix using the diagonal mean of the genetic variances estimated from 83 combinations and the off-diagonal using the mean genetic correlation, it does not form a positive definite matrix. Therefore, this study generated a genetic variance matrix for estimating the breeding value through the following process for producing a positive definite matrix from a genetic variance–covariance matrix [21].

(1) Genetic variance–covariance matrix was calculated using the mean of genetic parameters and mean correlation coefficients estimated from the initial variance component ($G_{0}$).

(2)$\;G_{0}$ was eigen-decomposed as follows.

$$G_{0}=Q\Lambda Q\prime$$

where, *Q* is an eigenvector, *Q′* is transpose of *Q* matrix and Λ is a diagonal matrix with eigenvalues.

(3) If an eigenvalue of Λ was a negative number, the eigenvalue was changed to 0.00001, close to 0, to create $\Lambda^{*}$.

(4) The genetic variance matrix to be used for the genetic capacity evaluation was calculated as follows in this study.

$$G_{0}=Q\Lambda^{*}Q\prime$$

The off-diagonal matrix of $G_{0}$ and that of G was negligible.

In this study, the BLUPF90 Program [24] of the BLUPF90 Family was used to estimate the breeding value.

### 2.4. Combining Estimated Breeding Values

The following equation was used to estimate the direct herd life (DHL) for the longevity trait by combining the breeding values for each parity and lactation while considering the mean survival rate of daughters for the sire estimated above [25]

$$s_{I}=\mathrm{mean}\;\mathrm{survival}\;\mathrm{rate}\;\mathrm{of}\;\mathrm{trait}\;i+\mathrm{sire}\;\mathrm{EVB}\;\mathrm{of}\;\mathrm{trait}\;i$$

And the cumulative survival rate of the trait i is

$$C_{i}=\overset{10}{\underset{i=1}{\prod }}S_{i}\;\mathrm{and}\;C_{10}=0$$

$$\mathrm{DHL}=\overset{10}{\underset{i=1}{\sum }}\left(C_{i-1}-C_{i}\right)\times \left(N_{i}+D_{i}\right)$$

where, DHL is the Direct herd life, $N_{i}$ is the calving interval of trait *i* (*N* = 0, first parity trait), and $D_{i}$ is the mean milking age for trait *i* until the culling of the individual.

In this study, the calving interval was calculated by setting the 1st–2nd calving as 426 days, the 2nd–3rd calving as 426 days, and the 3rd–4th calving as 432 days.

### 2.5. Comparing Breeding Value between Economic Lives, Production, and Conformation Trait

This study used the result of the national genetic evaluation published by the National Institute of Animal Science(NIAS) in April 2020 to compare breeding values between the longevity traits estimated above, milk production traits (adj-305 milk, adj-305 fat, adj-305 protein), those between 25 conformation traits (stature (ST), chest width (CW), body depth (BD), angularity (ANG), rump angle (RA), rump width (RW), rear leg side view (RLSV), foot angle (FA), fore udder attachment (FUA), rear udder height (RUH), rear udder width (RUW), udder suspensory (US), udder depth (UD), front teat placement (FTP), teat length (TL), overall conformation score (OCS), loin strength (LS), udder texture (UT), rear teat placement (RTP), heel depth (HD), bone quality (BQ), rear leg rear view (RLRV), height at front end (HFE), locomotion (Loc), and body condition score), and those between Udder Composite Index, Feet and Leg Composite, Milk, Fat, Protein composite, and Korean Type Production Index. This study used 383 sires with at least 50 daughters from DHL evaluation and 90% or higher reliability in EBV (adj-305 milk yield and OCS) for the accuracy of analysis (Supplementary Table S3).

## 3. Results and Discussion

### 3.1. Estimation of Genetic Parameters

The estimated genetic parameters including three fixed effects (herd-year, season and the first calving age) which were significantly affected by the survival period are presented in Table 1. The heritability estimated in this study ranged from 0.003 to 0.028, and the heritability tended to increase with an increase in parity. The heritability was estimated to be lowest (0.003, 0.004, and 0.006) in early-lactation (L1.1, L2.1, and L3.1, respectively) and 0.003, 0.007, and 0.014 in mid-lactation (L1.2, L2.2, and L3.2, respectively). In contrast, it was estimated as 0.022, 0.026, and 0.028 in late-lactation (L1.3, L2.3, and L3.3, respectively), which were the highest values for heritability of all. It is believed that the heritability in late-lactation (L1.3, L2.3, and L3.3) was higher than that in early-lactation and mid-lactation because the culling rate in late-lactation was much higher (62.5–80.7%) than that in early-lactation (6.0–10.2%) and mid-lactation (13.3–27.3%). The extreme phenotypic frequency in late-lactation may cause a bias [26]. However, Sasaki, et al. [27] also reported that heritability (0.042 ± 0.007~0.084 ± 0.007) increased toward the-late-lactation period of each parity in Japan. Heise, Liu, Stock, Rensing, Reinhardt and Simianer [21] also showed that heritability tended to increase (0.020 (L1.3)~0.026 (L3.3)) as parity increased, similar to the results of this study. The results obtained for heritability in late-lactation of all parities were similar to those in this study. However, the heritability in early- and mid-lactation of all parities ranged from 0.016 to 0.042, which was higher than that of this study. One of the possible reasons is the difference in the definition of lactation period. Moreover, the size of domestic dairy farms is around 50 head of cattle, which is smaller than the size of large dairy farms overseas. Therefore, it is easier to manage individuals in early- and mid-lactation. German VIT (https://www.vit.de/, accessed on 22 May 2020) reported that the culling rate was 17–24%, 31–35%, and 44–49% during early-, mid-, and late-lactation period, respectively. The culling rate in Korean Holstein was slightly different with 8–13%, 19–29% and 56–71% during early-, mid-, and late-lactation periods, respectively (Supplementary Figure S1).

Table 2 shows the genetic correlations among all traits. The results showed a strong genetic correlation for each lactation period with a mean of 0.19–0.99 and a standard deviation of 0.001–0.070 (0.63 ± 0.247). The mean genetic correlation between lactation periods in the same parity was lower than 0.9, except for a genetic correlation coefficient (0.94) between early-lactation (L1.1) and mid-lactation (L1.2) in the first parity ($r_{g_{1.1},g_{1.2}}$). Moreover, the genetic correlation coefficient between the same periods in the subsequent parity was estimated as 0.65–0.99, which was higher than that (0.19–0.85) between periods in the same parity. Additionally, the genetic correlation coefficient between the same period of the second and third parity was 0.96–0.99, which was higher than that (0.65–0.96) between the same period of the first and second parity. The last period, late-lactation, showed a strong correlation between 0.95 and 0.99. Heise [28] reported that the mean of all genetic correlations was less than 0.9, and the genetic correlation in early-lactation was between 0.082 and 0.096 in each parity. It was also reported that the genetic correlation for each lactation within the same parity was between 0.52 and 0.81. Additionally, it was reported that the genetic correlation coefficients between the second parity and third parity were higher than those between the first and second parities. Moreover, we identified strong genetic correlations (0.93~0.95) among the last lactation periods in all parities. Previous studies using multivariate analysis to evaluate survival traits suggested that survival in the first lactation could be genetically differentiated from survival in later lactation [9,10,11]. Considering that the range of the genetic correlation between the same lactation of the first parity and the second parity was between 0.65 and 0.96, which was lower than that between the second and third parity (0.96–0.99), this result could support those assumptions.

The multiple trait model is a method to utilize genetic and environmental correlations between traits by analyzing two or more traits simultaneously. The multiple trait model including traits with low heritability is useful when the difference between genetic and environmental correlations is high (e.g., r ≥ 0.5) or when the heritability of one trait is considerably higher than that of another trait [29]. In this study, when evaluating the genetic parameters estimated using uncensored data, low heritability and strong genetic correlations were estimated. Therefore, it was essential to use a multiple trait model. It is also believed that it will be possible to estimate the breeding value for the survival traits of individuals using censored data. For instance, although young bulls’ daughters are disadvantageous for evaluation because of a large amount of censored data, young bulls, which often have censored data, can be used for genetic evaluation using the characteristics of the multiple trait model.

It is not easy to directly compare the genetic parameters of longevity between countries because the lactation period is set differently between countries. Therefore, if the heritability and genetic correlations by parity are unified, it will be possible to compare countries directly and the results are shown in Table 3.

When cows survived in all three parities, heritability was estimated as 0.085, and it was 0.020, 0.028, and 0.039 in the first, second, and third parities, respectively. The heritability of this study was lower than that of Boettcher, et al. [30], and Heise, Liu, Stock, Rensing, Reinhardt and Simianer [21]. However, the genetic correlations of this study were similar to that of Boettcher, Jairath and Dekkers [30] and Heise, Liu, Stock, Rensing, Reinhardt and Simianer [21]. It is believed that heritability was estimated to be lower because of the differences in the feeding environment owing to the relatively small size of farms, considering that the heritability in late-lactation was similar but that in early- and mid-lactation was lower (Table 1).

### 3.2. Breeding Value Estimation and Breeding Value Correlation

The correlation between the DHL and the breeding value for longevity of each parity ranged between 0.76 and 0.96, showing a highly positive correlation. In all parities, the breeding value correlation between late lactation and DHL was always 0.9 or higher, which is a strong correlation (Table 4).

Sewalem, Miglior, Kistemaker, Sullivan, Huapaya and Van Doormaal [25] reported that the breeding value correlation of Canadian DHL with first calving to 120 days, 120–240 days, and 240 days to next calving was 0.978, 0.961, and 0.958, respectively. They also reported that the breeding value correlation of DHL with the second and third parities was 0.993 and 0.989, respectively, showing a strong correlation.

In this study, the correlation between early-lactation and mid-lactation in the first parity, early-lactation in the second parity and mid-lactation in the third parity was 0.9 or lower, which was different to the results of the previous studies. However, it could be explained by the differences in the culling rates in South Korea between early-, mid-, and late-lactation periods. It could also be because the heritability of early- and mid-lactation (0.003–0.014) was different to that of late-lactation (0.022–0.028). The analysis results of this study indicated that DHL included survival rates for all lactation periods. Moreover, it may be possible to compare different evaluation traits using these results.

In addition, this study analyzed the correlation of DHL with four traits related to milk production evaluated in South Korea, the breeding value of conformation traits, and four indices using these traits’ breeding value (Table 5 and Table 6A–C). The breeding value correlations of traits related to milk production with early- and mid-lactation were between 0.24 and 0.27, which was relatively high in the first parity. However, the range of breeding values after late lactation in the first parity was between −0.005 and 0.13, indicating that it was relatively low or negligible. Somatic Cell Score (SCS) showed a negative correlation with all lactation periods (−0.36 to −0.22). The breeding correlation between DHL and SCS increased as parity increased. The correlation between DHL and breeding values of milk production-related traits was weak (mostly between 0.13 and 0.15). However, the correlation with SCS was −0.31, which was similar to the breeding value by lactation period.

The results of Heise, Liu, Stock, Rensing, Reinhardt and Simianer [21] showed that the correlation between longevity and milk production traits ranged from 0.09 to 0.30. They also showed that the correlation with late-lactation was lower than that with early- and mid-lactation in all parities. However, in all parities, correlation with early- and mid-lactation, except for late-lactation, was 0.20 higher in previous studies, which is different to the results of this study. This could be because the culling rate in early- and mid-lactation were 17–24% and 31–35%, respectively, in Germany, which was higher than 6–10.2% and 13.3–27.3%, respectively, in South Korea. DHL and SCS showed the strongest negative correlation at mid-lactation (91–299 days), in the second and third parities, except for the first parity. Culling during this period is associated with milk production [14,31]. An association with mastitis, which is genetically correlated with SCS [32], was also found [14,33]. The different reasons for culling were consistent with previous studies showing a high frequency of breast-related diseases during mid-lactation. Previous studies that conducted multivariate analysis for survival-related traits showed that survival in the first lactation period was genetically related to later survival [9,11,30]. Therefore, it is believed that the results of this study can support these assumptions.

The breeding value correlation between DHL and conformation traits is shown in Table 6. 

The correlation between ST and breeding value by lactation was negative. Like milk production-related traits, it showed the highest breeding value correlation in early and mid-lactation of the first parity. However, the correlation between these traits was negative. The breeding value correlations of lactation with CW, BD, AN, and RW were negative (−0.44 to −0.05). CW and BD showed a moderate negative correlation (−0.43 to −0.34) in all parities. ANG also showed a negative correlation in all periods. In particular, correlations in late-lactation showed relatively stronger negative relationships. Similar to ANG, RW also showed a relatively high negative correlation in late-lactation of each parity. FA showed a strong breeding value correlation with early-lactation in most parities.

Breeding value correlations of traits related to udders mostly showed moderate positive correlations. In FUA, early-lactation in all parities was the highest with 0.10, 0.17, and 0.14, respectively, and the breeding value in mid-lactation had the strongest correlation. RUH, US, and UD showed positive correlations ranging from 0.22 to 0.52. In particular, UD was 0.36–0.52, showing a moderate correlation. In addition, correlation tended to be stronger in later lactation and later parity.

In particular, the results of this study revealed that the breeding value correlations with TL, OCS, and LS, which South Korean dairy farmers were keen on understanding, were not high (−0.10 to 0.13), unlike the expectations previous to the study. LS showed a negative correlation in the range of −0.12 to 0.07.

Moreover, UT was 0.28 (L1.1), 0.26 (L1.2), 0.14 (L2.1), 0.20 (L2.2), and 0.10 (L3.1) in early- and late- lactation of each parity. In late-lactation, the correlations of the first parity, second parity, and third parity were low with −0.01, −0.01, and 0.03, respectively. In RTP, which farmers paid much attention to during milking, there were positive correlations in all periods, but they were weak (0.04–0.12). HFE showed a negative correlation (−0.35 to −0.29) in all parities and lactation periods.

This study calculated the breeding value correlations between longevity-related DHL by applying UDC, FLC, MFP, and KTPI estimated by combining the breeding value estimated from Korea Genetic Capacity Evaluation (Table 7). 

As shown above, along with the results showing relatively stronger positive correlations with udder-related traits, the udder index calculated by combining udder traits showed intermediate correlations in the range of 0.30–0.44. In addition, as parity increased, the correlation between the breeding value and the udder index tended to increase from 0.29 to 0.44. FLC also showed a positive correlation. In the case of MFP, relatively high breeding value correlations were found in early- and mid-lactation of the first parity, similar to milk production traits. Lastly, KTPI, composited using milk production traits and body shape-related traits, showed positive correlations (0.25–0.36) in early- and mid-lactation of all parities with a small culling rate.

The breeding value correlation between DHL and conformation-related traits revealed that the correlations with RUH, US, and UD among traits-related to udder were relatively high (0.25– 0.52). In particular, the correlation with UD was the highest at 0.52. It appeared to be related to the heritability of udder-related traits associated with milk production after calving to the subsequent breeding. It is possible to improve the longevity of the individuals by improving these traits.

In contrast, the results of this study showed that longevity had a negative correlation with traits directly related to body size (STA, CW, BD, ANG, and HFE). Specht, et al. [34] reported that the phenotypic correlation between the first linear examination score and longevity was 0.2. Among the relationships between 66 conformation traits and longevity measured in the first lactation using the information on sires’ daughters, RTP (0.38), important traits (0.35), and US (0.26) were strongly correlated with longevity [35]. The US of this study was 0.25, similar to the previous study. Although it was slightly lower, RTP (0.11) was also similar to that observed in previous studies.

Vacek, et al. [36] reported that herd life and body shape traits had a generally weak phenotypic correlation (−0.048 to 0.132). Among them, ANG, ST, CW, and BD were 0.094, 0.087, 0.065, and 0.040, respectively, which were positive correlations that disagreed with the results of this study. However, other traits were similar or lower than the results of this study.

DHL showed positive correlations (0.14–0.42) with UDC, FLC, MFP, and KTPI, calculated by combining these traits. It is possible to, directly and indirectly, select the longevity of Korean dairy cattle using these indices.

In addition, this study analyzed both censored and uncensored data. The results of this study will provide baseline information for estimating life expectancy solely by using censored data.

## 4. Conclusions

This study presented evidence that individuals’ genetic backgrounds differed with respect to differences in the individuals’ survival period. It provides the basis for developing a more accurate genetic evaluation model for traits related to longevity that will coherently explain the genetic correlation structure. It will also be possible to provide information on the economic aspects to Korean dairy farmers by comparing breeding value between milk production-related traits, conformation-related traits, and reproduction-related traits.

DHL and each lactation period showed a strong correlation. Milk production traits, udder-related traits, and composite indices (UDC, FLC, MFP, and KTPI) had positive correlations. The results indicate that these traits are closely related to the longevity and survival of cows. It is believed that it will be possible to select a sire based on these traits.

The results of this study may serve as the basis for developing a more accurate model for evaluating longevity traits in South Korea.

## Figures and Tables

**Table 1:** Additive variance, phenotype variance, heritability, and number of contributing runs (*n*).

Trait *	*n*	Additive Variance		Phenotype Variance		Heritability	
		Mean	S.D.	Mean	S.D.	Mean	S.D.
1.1	27	0.000083	0.000004	0.025140	0.000001	0.003	0.000
1.2	28	0.000157	0.000017	0.047193	0.000153	0.003	0.000
1.3	28	0.003129	0.000314	0.139371	0.000115	0.022	0.002
2.1	28	0.000117	0.000013	0.030623	0.000036	0.004	0.000
2.2	28	0.000543	0.000044	0.073981	0.000163	0.007	0.001
2.3	28	0.003783	0.000381	0.148158	0.000131	0.026	0.003
3.1	28	0.000242	0.000033	0.041053	0.000026	0.006	0.001
3.2	27	0.001269	0.000064	0.088283	0.000112	0.014	0.001
3.3	27	0.004590	0.000845	0.166806	0.000253	0.028	0.005

* The lactation period was divided into early-lactation (0–90 days: L1.1, L2.1, and L3.1), mid-lactation (91–299 days: L1.2, L2.2, and L3.2), and late-lactation (300 days—next parity: L1.3, L2.3, and L3.3). S.D.: Standard Deviation.

**Table 2:** Genetic correlation estimates with means, standard deviation, and number of contributing runs (*n*).

Trait *	Variable	1.2	1.3	2.1	2.2	2.3	3.1	3.2	3.3
1.1	Mean	0.94	0.19	0.77	0.65	0.25	0.48	0.71	0.16
	S.D.	0.006	0.016	0.042	0.031	0.047	0.049	0.033	0.032
	*n*	7	7	7	7	7	7	6	6
1.2	Mean		0.42	0.78	0.65	0.26	0.72	0.67	0.34
	S.D.		0.041	0.045	0.024	0.044	0.062	0.032	0.034
	*n*		7	7	7	7	7	7	7
1.3	Mean			0.19	0.57	0.96	0.65	0.50	0.95
	S.D.			0.070	0.038	0.004	0.047	0.020	0.004
	*n*			7	7	7	7	7	7
2.1	Mean				0.85	0.52	0.96	0.30	0.41
	S.D.				0.046	0.038	0.037	0.026	0.066
	*n*				7	7	7	7	7
2.2	Mean					0.67	0.92	0.99	0.68
	S.D.					0.014	0.043	0.042	0.028
	*n*					7	7	7	7
2.3	Mean						0.85	0.51	0.99
	S.D.						0.024	0.021	0.002
	*n*						7	7	7
3.1	Mean							0.57	0.77
	S.D.							0.021	0.036
	*n*							7	7
3.2	Mean								0.71
	S.D.								0.045
	*n*								6

* The lactation period was divided into early-lactation (0–90 days: L1.1, L2.1, and L3.1), mid-lactation (91–299 days: L1.2, L2.2, and L3.2), and late-lactation (300 days—next parity: L1.3, L2.3, and L3.3). S.D.: Standard Deviation.

**Table 3:** Genetic correlation (upper diagonal) and phenotypic correlation (below diagonal), heritability (diagonal) per parity.

Parity	1	2	3
1	0.020	0.877	0.873
2	−0.027	0.028	0.947
3	−0.010	−0.043	0.039

**Table 4:** Correlations among the nine traits and direct herd life for Korean Holstein.

Trait *	1.1	1.2	1.3	2.1	2.2	2.3	3.1	3.2	3.3
DHL	0.76	0.82	0.90	0.74	0.92	0.90	0.96	0.87	0.92

* DHL: Direct herd life.

**Table 5:** Breeding value correlations among the milk production traits and sires’ direct herd life for Korean Holsteins.

Traits *	Milk	Fat	Protein	SCS
1.1	0.26	0.27	0.27	−0.22
1.2	0.25	0.24	0.24	−0.22
1.3	0.01	0.03	0.03	−0.25
2.1	0.12	0.15	0.11	−0.27
2.2	0.13	0.19	0.13	−0.36
2.3	−0.005	0.02	0.01	−0.28
3.1	0.08	0.12	0.08	−0.33
3.2	0.13	0.17	0.12	−0.35
3.3	0.01	0.04	0.02	−0.29
DHL	0.13	0.15	0.13	−0.31

* DHL: direct herd life; Milk: milk yield; Fat: milk fat; Protein; milk protein; SCS: somatic cell score.

**Table 6:** (**A**) Breeding value correlations among the conformation traits and sires’ direct herd life for Korean Holstein. (**B**) Breeding value correlations among the conformation traits and sires’ direct herd life for Korean Holstein. (**C**) Breeding value correlations among the conformation traits and sires’ direct herd life for Korean Holstein.

(A)									
Traits	Stature	Chest Width	Body Depth	Angularity	Rump Angle	Rump Width	Rear Leg Side View		Foot Angle
1.1	−0.21	−0.40	−0.36	−0.12	−0.06	−0.14	0.04		0.19
1.2	−0.25	−0.40	−0.37	−0.12	−0.03	−0.14	−0.02		0.17
1.3	−0.14	−0.39	−0.42	−0.26	0.14	−0.25	−0.06		0.08
2.1	−0.13	−0.34	−0.34	−0.11	−0.02	−0.12	0.04		0.17
2.2	−0.16	−0.43	−0.41	−0.09	0.04	−0.17	0.00		0.09
2.3	−0.10	−0.39	−0.42	−0.23	0.14	−0.24	−0.04		0.09
3.1	−0.15	−0.43	−0.44	−0.18	0.07	−0.20	−0.01		0.14
3.2	−0.13	−0.43	−0.40	−0.05	0.05	−0.17	0.00		0.06
3.3	−0.10	−0.41	−0.43	−0.20	0.13	−0.24	−0.03		0.07
Direct Herd Life	−0.19	−0.47	−0.47	−0.21	0.07	−0.24	−0.02		0.14
**(** **B)**									
**Traits**	**Fore Udder Attachment**	**Rear Udder Height**	**Rear Udder Width**	**Udder Suspensory**	**Udder Depth**	**Front Teat Placement**	**Front Teat Length**		**Overall Conformation Score**
1.1	0.10	0.22	−0.19	0.19	0.36	−0.11	−0.06		0.08
1.2	0.11	0.22	−0.17	0.19	0.36	−0.13	−0.06		0.08
1.3	0.06	0.27	−0.35	0.19	0.45	−0.09	−0.05		0.00
2.1	0.17	0.23	−0.24	0.26	0.42	−0.10	0.00		0.13
2.2	0.14	0.29	−0.31	0.27	0.50	−0.10	−0.08		0.12
2.3	0.08	0.29	−0.38	0.22	0.49	−0.09	−0.05		0.03
3.1	0.14	0.30	−0.34	0.28	0.52	−0.11	−0.04		0.10
3.2	0.13	0.29	−0.31	0.27	0.50	−0.10	−0.10		0.12
3.3	0.09	0.30	−0.38	0.24	0.51	−0.09	−0.06		0.04
Direct Herd Life	0.11	0.30	−0.35	0.25	0.52	−0.12	−0.06		0.06
**(** **C)**									
**Traits**	**Loin Strength**	**Udder Texture**	**Rear Teat Placement**	**Heel Depth**	**Bone Quality**	**Rear Leg Rear View**	**Height at Front End**	**Locomotion**	**Body Condition Score**
1.1	−0.10	0.28	0.04	0.24	0.13	0.05	−0.35	0.19	−0.11
1.2	−0.11	0.26	0.09	0.22	0.14	0.07	−0.37	0.20	−0.11
1.3	−0.13	−0.01	0.10	0.12	0.07	0.04	−0.30	0.09	−0.02
2.1	−0.07	0.14	0.11	0.18	0.13	0.11	−0.29	0.16	−0.11
2.2	−0.10	0.20	0.08	0.15	0.25	0.01	−0.34	0.13	−0.16
2.3	−0.12	−0.01	0.11	0.11	0.09	0.04	−0.29	0.09	−0.04
3.1	−0.11	0.10	0.12	0.17	0.16	0.08	−0.34	0.14	−0.10
3.2	−0.10	0.22	0.07	0.13	0.29	−0.03	−0.31	0.11	−0.19
3.3	−0.12	0.03	0.10	0.11	0.14	0.02	−0.29	0.08	−0.07
Direct Herd Life	−0.14	0.13	0.11	0.19	0.14	0.06	−0.38	0.16	−0.09

**Table 7:** Breeding value correlations among the composite index and sires’ direct herd life for Korean Holstein.

Traits *	UDC	FLC	MFP	KTPI
1.1	0.30	0.16	0.29	0.36
1.2	0.29	0.16	0.26	0.33
1.3	0.35	0.09	0.03	0.13
2.1	0.37	0.17	0.16	0.27
2.2	0.42	0.07	0.19	0.30
2.3	0.39	0.09	0.02	0.13
3.1	0.44	0.14	0.13	0.25
3.2	0.42	0.03	0.18	0.28
3.3	0.41	0.07	0.04	0.14
DHL	0.42	0.14	0.27	0.16

* UDC: udder composite index; FLC: feet and leg composite; MFP: milk, fat and protein composite; KTPI: Korean type production index; DHL: direct herd life.

## Data Availability

The data presented in this study are available on request from the corresponding author.

## Supplementary Materials

The following supporting information can be downloaded at: https://www.mdpi.com/article/10.3390/ani12060701/s1, Figure S1: South Korea culling rate by lactation and parity; Table S1: Number of culls by year and culling reasons with uncensored data; Table S2: Definition of each periods and parity for survival trait; Table S3: Basic statistics of sires which is result of national evaluation from NIAS in April 2020.
