# Supplementary material for: The Estimation of Genetic Parameters for Longevity According to Lactation Period Using a Multiple Trait Animal Model in Korean Holstein Cows
Source: Animals (Basel). 2022 Mar 11;12(6):701. doi: 10.3390/ani12060701 (PMC8944745; doi:10.3390/ani12060701)
Supplement: Supplementary file 1 [file animals-12-00701-s001.zip › animals-1458924-supplementary.pdf]

Supplementary Table S1. Number of culls by year and culling reasons with uncensored data.

Culling reason	Culling Year													
	~ 2015		2015		2016		2017		2018		2019		Total	
	N	%	N	%	N	%	N	%	N	%	N	%	N	%
ETC.	59,534	26	17,682	57	15,853	54	14,640	53	14,505	51	11,396	45	133,610	32.1
Missing	96,772	42	280	1	7	0	10	0	10	0	-	0	97,079	23.3
Sickness	19,318	8	2,848	9	2,942	10	2847	10	3090	11	3507	14	34,552	8.3
Low milk production	16,129	7	3,663	12	3,330	11	3399	12	3420	12	3215	13	33,156	8
Reproductive	17,529	8	2,786	9	2,595	9	2694	10	3014	11	3146	12	31,764	7.6
Mastitis	11,441	5	1,896	6	2,294	8	2144	8	2525	9	2185	9	22,485	5.4
Injury	5318	2	940	3	1,097	4	1008	4	803	3	712	3	9878	2.4
Feet and leg problems	2055	1	567	2	507	2	521	2	389	1	370	1	4409	1.1
Old age	974	0	181	1	193	1	173	1	231	1	218	1	1970	0.5
Slow milker	233	0	105	0	169	1	101	0	352	1	347	1	1307	0.3
Milk fever	613	0	39	0	41	0	72	0	90	0	73	0	928	0.2
Displaced abomasum	617	0	64	0	45	0	40	0	52	0	57	0	875	0.2
Injury to udder	526	0	78	0	40	0	43	0	78	0	44	0	809	0.2

Bloat	429	0	45	0	43	0	55	0	67	0	90	0	729	0.2
Poison	78	0	4	0	9	0	5	0	4	0	5	0	105	0.03
Electrocution	34	0	2	0	6	0	5	0	-	0	5	0	52	0.01
Total	231,600	100	31,180	100	29,171	100	27,757	100	28,630	100	25,370	100	373,708	100

Table S2. Definition of each periods and parity for survival trait.

**Lactation**	**Period**	**Trait**	**Start (day from calving)**	**End (day from calving)**
**1**	**1**	L1.1	0	90
1	2	L1.2	91	299
1	3	L1.3	300	2nd calving
2	1	L2.1	0	90
2	2	L2.2	91	299
2	3	L2.3	300	3rd calving
3	1	L3.1	0	90
3	2	L3.2	91	299
3	3	L3.3	300	4th calving

Table S3. Basic statistics of sires which is result of national evaluation from NIAS in April, 2020.

	N	Mean	S.D.	Min.	Max.
Number of daughters*	383	642.98	698.72	52	4674
Reliability of EBV for milk yield	383	96.78	1.67	92	99
Reliability of EBV for overall conformation score	383	94.38	2.62	90	99

Number of daughters is used for DHL estimation; NIAS: National Institute of Animal Science

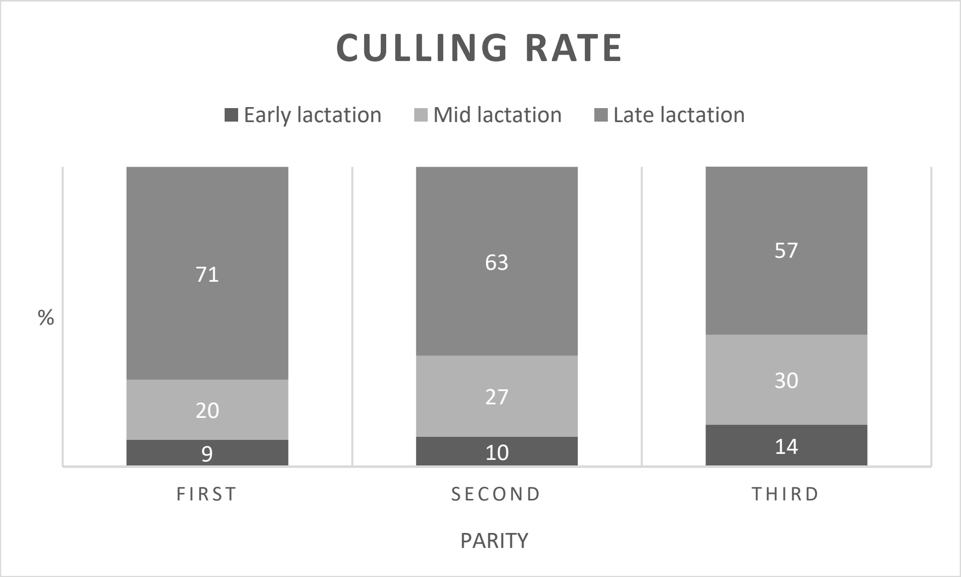

Figure S1. South Korea culling rate by lactation and parity.
